# Surprise Finding of Uterine Torsion During a Routine Scheduled Repeat Cesarean Section: A Case Report

**DOI:** 10.1155/crog/5529772

**Published:** 2025-11-17

**Authors:** Asma Saleem, Dorothy Charles, Jason Lowe, Sarah E. Donohue

**Affiliations:** ^1^Department of Family & Community Medicine, University of Illinois College of Medicine Peoria, Peoria, Illinois, USA; ^2^Department of Psychiatry and Behavioral Medicine, University of Illinois College of Medicine Peoria, Peoria, Illinois, USA

**Keywords:** case report, cesarean section, macrosomia, maternal morbidity, surgical complication, term pregnancy complication, uterine rotation, uterine torsion

## Abstract

**Introduction:**

This article describes a case of asymptomatic uterine torsion in the setting of macrosomia and polyhydramnios. While other causes have been reported in the literature, there are no existing cases of uterine torsion associated with fetal macrosomia and polyhydramnios.

**Case:**

This patient had an asymptomatic uterine torsion discovered incidentally during a scheduled repeat cesarean section. A congested vascular lower uterine segment was noticed on entrance into the abdomen. After exteriorization of the uterus, 180° torsion was discovered. If torsion is identified prior to hysterotomy, an attempt can be made to perform detorsion. If unsuccessful, a posterior hysterotomy can be performed to avoid the bilateral uterine vessels. In our case, the torsion was not discovered until after exteriorization of the uterus, so an unintended posterior classical (vertical) uterine hysterotomy was performed to avoid the bilateral uterine vessels.

**Conclusion:**

While prior cases of uterine torsion have had other etiologies for increased size of the gravid uterus, including large fibroids, twins, and malpresentation, the likely cause in this case was fetal macrosomia and polyhydramnios. Uterine torsion should be considered as part of the differential diagnosis for abdominal and pelvic pain in pregnancy, especially with any risk factors, although it may be asymptomatic. Ultrasound, MRI, and, in select cases, CT (outside of pregnancy) can be helpful imaging modalities to evaluate for uterine torsion, but it is often not detected. The finding of a congested vascular lower uterine segment should raise the surgeon's index of suspicion for uterine torsion. Surgical management of uterine torsion in pregnancy includes reducing the torsion when possible or performing a posterior hysterotomy when attempts at reduction are not successful. Prophylactic shortening of the round ligament after delivery may be considered to prevent recurrence, but this technique is not yet validated.

**Key Points**

• An increased size of the gravid uterus, such as is found with fibroids or twins, including with macrosomia or polyhydramnios, should be considered risk factors for uterine torsion.
• Uterine torsion should be considered as part of the differential diagnosis for abdominal and pelvic pain in pregnancy.
• Ultrasound, MRI, and, in select cases, CT (outside of pregnancy) can be helpful and appropriate imaging modalities to evaluate for uterine torsion.
• Surgical management of uterine torsion in pregnancy includes reducing the torsion when possible, performing a posterior hysterotomy when attempts at reduction are not successful, and consideration of prophylactically shortening the round ligament after delivery to prevent recurrence.
• The finding of a congested vascular lower uterine segment should raise the surgeon's index of suspicion for uterine torsion.

## 1. Introduction

Uterine torsion is a rare gynecological condition in which the uterus is rotated at least 45° [1, 2]. It is most often seen in pregnancy due to the increased weight of the uterus and ligamentous laxity. Risk factors for uterine torsion include anything causing a large or misshapen uterus and cases have mostly been seen with large fibroids. When uterine torsion occurs during pregnancy, the symptoms are often nonspecific, which makes it difficult to diagnose preoperatively.

Here, we describe a case where during a scheduled repeat cesarean section, an incidental uterine torsion was found and an unintended posterior hysterotomy was done at the time of delivery. The patient had macrosomia and polyhydramnios, both of which could have contributed to the risk for uterine torsion.

## 2. Case

A 27-year-old Gravida 3, Para 1, female presented to the delivery ward at 38 weeks and 1 day of gestation for a scheduled repeat cesarean section. Her pregnancy was complicated by Type 2 diabetes mellitus with a history of a previous macrosomic infant (4649 g) requiring cesarean delivery, and her prenatal care was managed in consultation with maternal–fetal medicine (MFM). During this pregnancy, she had suboptimal glucose control on insulin therapy and was found to also have polyhydramnios and fetal macrosomia. Of note, she had a normal body mass index (BMI) prior to and during pregnancy. The course of her pregnancy was otherwise uneventful. The patient's only complaint was of mild back pain typical of pregnancy. Given her overall clinical picture, early term delivery was recommended by MFM. An ultrasound performed 1 day prior to the scheduled cesarean section showed an estimated fetal weight of 5647 g, which was above the 99th percentile for gestational age.

**Figure 1:**
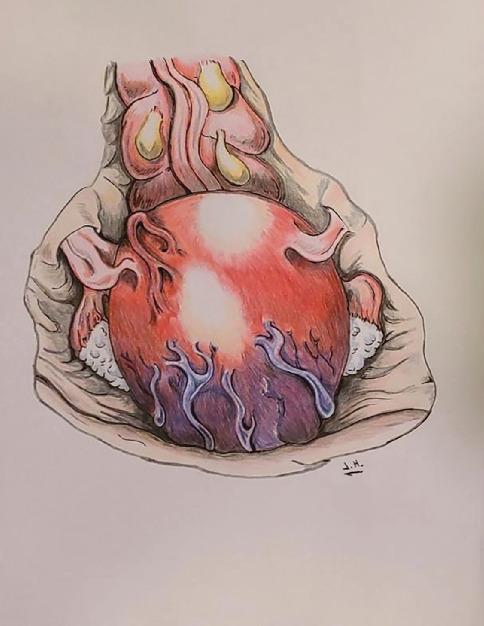
Artistic rendering of the uterus upon entry into the abdomen, notable for tortuous vessels visible in the lower uterine segment.

On the day of surgery, the patient was feeling well without acute complaints. The surgery proceeded as planned. Upon entry into the abdominal cavity, the entire lower uterine segment was notable for overlying engorged and tortuous uterine vessels (see Figure 1 for illustration), which were concerning for possible placental vasculature visible through a uterine window. To avoid hemorrhage, a higher vertical (classical) uterine incision was made. Amniotomy was performed for clear fluid, and a viable macrosomic infant was delivered with vacuum assistance from a cephalic presentation. The placenta was delivered without difficulty.

**Figure 2:**
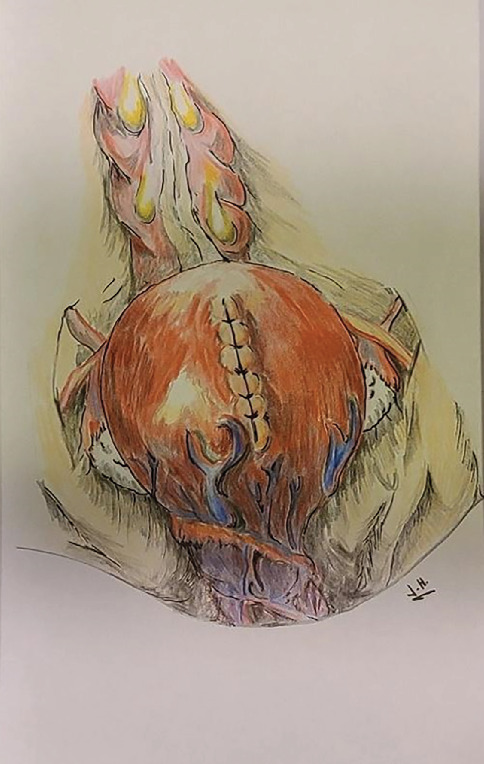
Artistic rendering of the unintentional vertical posterior hysterotomy site after repair and prior to detorsion.

**Figure 3:**
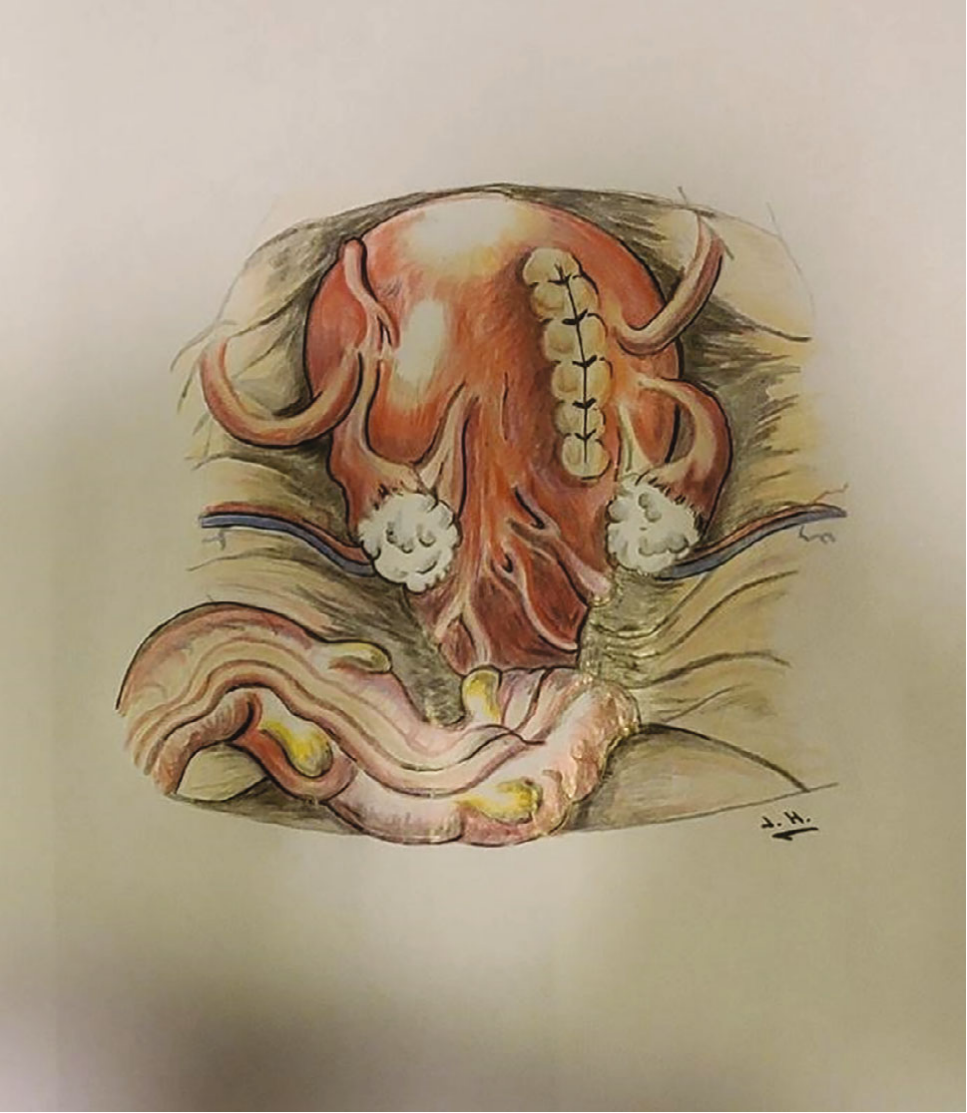
Artistic rendering of the posterior view of the repaired uterus after detorsion.

The uterus was then exteriorized from the abdomen. At this point, it was noted to be rotated 180° clockwise on its longitudinal axis. Therefore, the hysterotomy was done unintentionally on the posterior aspect of the uterus. The uterus was kept in this rotated position until closure of the hysterotomy (see Figure 2 for illustration). It was then manually rotated counterclockwise back into the normal anatomic position. The posterior aspect of the uterus was examined, which demonstrated the vertical incision on the right side (see Figure 3 for illustration).

The infant was healthy with a birth weight of 5669 g. The remainder of the patient's postoperative course was uncomplicated.

Prior to discharge, the team discussed the risks associated with a posterior and a vertical uterine scar, particularly for a close interval pregnancy. She was also counseled that the recommendation for future pregnancies would be delivered by repeat cesarean section between 36 and 37 weeks' gestation.

**Figure 4:**
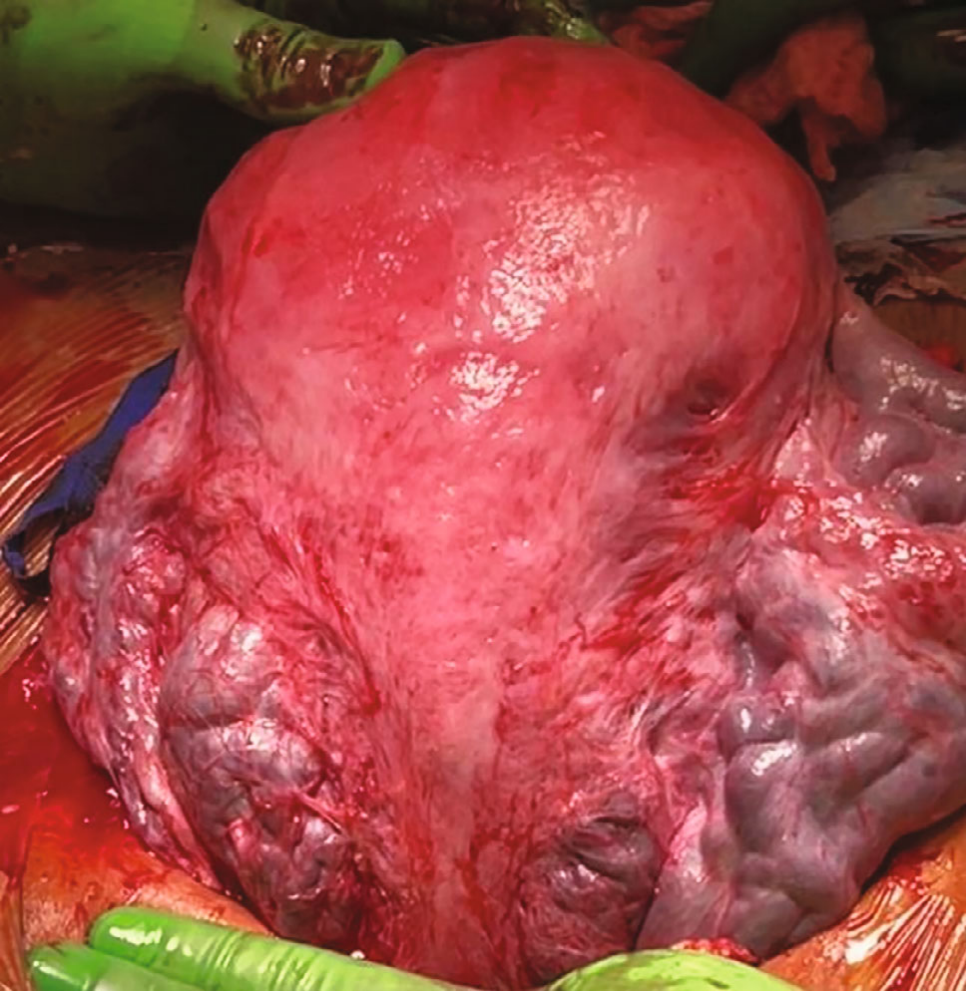
Intraoperative photograph of the posterior aspect of the uterus during the subsequent cesarean section. Note the suspected scar from the previous surgery on the right.

Interestingly, this patient presented 2 years later with another pregnancy complicated again by fetal macrosomia. MFM recommended delivery at 37 weeks' gestation. She did not have recurrence of uterine torsion. After uterine closure was completed, the posterior aspect of the uterus was examined. She had excellent scar healing, and it was difficult to identify the scar from the previous uterine incision (Figure 4).

## 3. Discussion

Uterine torsion during pregnancy, while uncommon, can be dangerous to both the birthing individual and their fetus. Most cases of uterine torsion in pregnancy, at or near term, have led to emergency cesarean births, ultimately confirming diagnosis. Cases of uterine rotation of up to 720° have been documented [3]. While it is rare, uterine torsion has been described in a wide range of ages and during all trimesters of pregnancy [4]. Risk factors include anything causing a large or misshapen uterus. Most often, this has been seen with large fibroids [5]. It has also been associated with Müllerian anomalies [2, 6], fetal malpresentation (particularly transverse lie) [2], pelvic adhesions, and abdominal or ligamentous laxity [2]. A case has been seen with a twin pregnancy [7] and another in the setting of Ehlers–Danlos syndrome [8]. These all cause the uterus to be enlarged, be rotated, or have additional ligamentous laxity. Our case further suggests that both fetal macrosomia and polyhydramnios should be considered risk factors for this condition, as they cause enlargement of the uterus.

Uterine torsion is associated with increased peripartum morbidity and notable mortality rates of up to 12% [2, 9]. Peripartum risks include pain, cesarean section, hematoperitoneum, placental abruption, postpartum hemorrhage, and uterine atony and can lead to ischemia, shock, or disseminated intravascular coagulation [2, 4, 9]. Additionally, recurrence in subsequent pregnancies has been documented in two case reports [10], with two additional case reports documenting a successful delivery in a subsequent pregnancy [11, 12]. Fetal risks include growth restriction, nonreassuring fetal well-being, and even fetal death from ischemia [2, 4, 13].

Clinical presentation of torsion often includes arrest of labor, abdominal pain, vaginal bleeding, nausea or vomiting, shock, dizziness, or urinary or gastrointestinal symptoms [2, 13–15]. However, in up to 11% of cases, it may be asymptomatic [2, 16]. The finding of torsion in our patient was particularly surprising as she had no concerning symptoms throughout pregnancy. As in her case, seemingly normal pregnancy-related concerns such as abdominal and back pain may be the only presenting symptom, and uterine torsion should remain on the differential, especially when known risk factors are present.

Ideally, cases of uterine torsion can be identified preoperatively, but the symptoms are often nonspecific, and typically, diagnosis is only made during surgery. Often, surgery is done emergently in the setting of shock, severe pain, or fetal distress. When suspected, anatomy can be assessed using imaging modalities such as ultrasound and MRI [17] and CT in nonpregnant individuals [18]. However, it is important to remember that torsion is not always recognized with imaging, and it may also be incorrectly diagnosed. One case report had a diagnosis of uterine incarceration on ultrasound prior to delivery, which then turned out to be uterine torsion [17]. Another case showed placental abruption on ultrasound, which could have obscured the true finding of torsion [4]. Other changes noticed on ultrasound have been a change in the placental location; however, the authors only identified the uterine torsion upon cesarean section and only in hindsight noted that the change seen on the ultrasound may have been an indication [19]. In our case, the ultrasound did not report any concerning findings such as torsion. Changes in the size and shape of fibroids [18] or the location of the placenta from prior ultrasounds [4] should also prompt consideration of torsion.

Regardless of preoperative suspicion, the finding of a vascular lower uterine segment during cesarean section should raise the index of suspicion for uterine torsion and prompt the surgeon to pause [16]. Ovarian vessels covering the posterior lower uterine wall may appear similarly to placental vasculature in a thin lower uterine segment. In our case, the vessels were thought to be placental but then after delivery it was revealed they were ovarian.

Treatment of uterine torsion can be done through manual detorsion during laparotomy [1] or cesarean delivery [18]. This involves rotating the uterus around its longitudinal axis back to its anatomical position. In pregnancy, confirming torsion prior to hysterotomy would provide an opportunity to attempt to detort the uterus prior to delivery of the fetus. Only when an attempt at detorsion is unsuccessful would a deliberate posterior hysterotomy be necessary [18, 20, 21]. Because we did not identify the torsion until after the delivery of the infant, we unintentionally performed a posterior vertical uterine incision. In our case, the detorsion of the uterus was done after the repair of the hysterotomy.

Depending on the degree of uterine torsion, the surgeon may consider shortening of the round ligaments at this time to prevent recurrence in the postpartum period or with future pregnancies, although this has not been validated [9, 22].

## 4. Conclusion

It is important to consider uterine torsion as an obstetric complication when thinking about causes of acute abdominal or pelvic pain in pregnancy. Our finding was particularly surprising as the patient was asymptomatic throughout pregnancy. If upon entering the abdomen, a congested lower uterine segment is noted, uterine torsion should be suspected, as these can be ovarian vessels covering the posterior lower uterine wall. An attempt to detort the uterus should be made before the delivery of the fetus. If unsuccessful, a posterior hysterotomy can be made to deliver the fetus. In our case, the posterior hysterotomy was unintentional as the torsion was not identified until after delivery. The etiology of torsion in our case was most likely macrosomia and polyhydramnios causing the enlarged uterus, which has not been reported in torsion cases previously. We suggest that macrosomia or polyhydramnios should be considered risk factors for uterine torsion.

## Data Availability

Data sharing is not applicable to this article as no datasets were generated or analyzed during the current study.

## References

[1] Chua K. J., Patel R., Eana A., Varughese J. (2019). Uterine Torsion With Necrosis of Bilateral Adnexa in a Postmenopausal Woman. *BML Case Reports*.

[2] Jensen J. G. (1992). Uterine Torsion in Pregnancy. *Acta Obstetricia Et Gynecologica Scandinavica*.

[3] Kilicci C., Sanverdi I., Bostanci E., Abide C. Y., Eser S. K. (2018). Uterine Torsion of 720 Degrees in the Third Trimester of Pregnancy and Accompanying Bladder Torsion: A Case Report. *Pan African Medical Journal*.

[4] Zullino S., Faiola S., Paganelli A. M., Ferrazzi E. (2014). A Case of Abruptio Placentae due to the Torsion of Gravid Uterus. *Case Reports in Obstetrics and Gynecology*.

[5] Deshpande G., Kaul R., Manjuladevi P. (2011). A Case of Torsion of Gravid Uterus Caused by Leiomyoma. *Case Reports in Obstetrics and Gynecology*.

[6] Demaria F., Goffinet F., Jouannic J. M., Cabrol D. (2005). Preterm Torsion of a Gravid Uterus Didelphys Horn of a Twin Pregnancy. *Obstetrics and Gynecology*.

[7] Thubert T., Abdul Razak R., Villefranque V., Muray J. M., Picone O., Deffieux X. (2011). Uterine Torsion in Twin Pregnancy. *Journal of Gynecology Obstetrics and Human Reproduction*.

[8] Ghalandarpoor-Attar S. N., Ghalandarpoor-Attar S. M. (2022). Uterine Torsion as an Elusive Obstetrical Emergency in Pregnancy: Is There an Association Between Gravid Uterus Torsion and Ehlers-Danlos Syndrome?: A Case Report. *Journal of Medical Case Reports*.

[9] Liang R., Gandhi J., Rahmani B., Ali K. S. (2020). Uterine Torsion: A Review With Critical Considerations for the Obstetrician and Gynecologist. *Translational Research in Anatomy*.

[10] Corr J. (1943). Axial Torsion of the Gravid Uterus in Two Successive Pregnancies. *American Journal of Obstetrics and Gynecology*.

[11] Boehm K., Gheissari M., Crownover D., Frugoli A. (2024). Do Not Get Your Uterus Twisted: A Case Report of a 180-Degree Torsion of Term Gravid Uterus and a Review of the Literature. *Cureus*.

[12] Fatih F. F., Gowri V., Rao K. (2012). Uterine Torsion in Second Trimester of Pregnancy Followed by a Successful-Term Pregnancy. *BML Case Reports*.

[13] Cook K. E., Jenkins S. M. (2005). Pathologic Uterine Torsion Associated With Placental Abruption, Maternal Shock, and Intrauterine Fetal Demise. *American Journal of Obstetrics and Gynecology*.

[14] Moores K. L., Wood M. G., Foon R. P. (2014). A Rare Obstetric Emergency: Acute Uterine Torsion in a 32-Week Pregnancy. *BML Case Reports*.

[15] Wilson D., Mahalingham A., Ross S. (2006). Third Trimester Uterine Torsion: Case Report. *Journal of Obstetrics and Gynaecology Canada*.

[16] Arumugham S., Mathew M., Deoskar S., Sharma J. (2013). Uterine Torsion Mimicking Supine Hypotension Syndrome After Regional Anaesthesia. *BML Case Reports*.

[17] Oda H., Yamada Y., Uehara Y. (2020). Uterine Torsion in an Elderly Woman Associated With Leiomyoma and Continuously Elevating Muscle Enzymes: A Case Study and Review of Literature. *Case Reports in Obstetrics and Gynecology*.

[18] De Ioris A., Pezzuto C., Nardelli G. B., Modena A. B. (2010). Caesarean Delivery Through Deliberate Posterior Hysterotomy in Irreducible Uterine Torsion: Case Report. *Acta Bio-Medica*.

[19] Kremer J. A., van Dongen P. W. (1989). Torsion of the Pregnant Uterus With a Change in Placental Localization on Ultrasound; a Case Report. *European Journal of Obstetrics, Gynecology, and Reproductive Biology*.

[20] Albayrak M., Benian A., Ozdemir I., Demiraran Y., Guralp O. (2011). Deliberate Posterior Low Transverse Incision at Cesarean Section of a Gravid Uterus in 180 Degrees of Torsion: A Case Report. *Journal of Reproductive Medicine*.

[21] Makwe C. C., Ugwu A. O., Osuji G. N., Anumni C., Ekor O. E., Asiyanbi G. K. (2021). Posterior Classical Caesarean Section in a Myomatous Gravid Uterus at Term: A Case Report. *International Journal of Surgery Case Reports*.

[22] Pelosi M. A., Pelosi M. A. (1998). Managing Extreme Uterine Torsion at Term. A Case Report. *Journal of Reproductive Medicine*.

